# Full Deep Breathing

**Published:** 1897-08

**Authors:** 


Professor Bellal says: "Enough cannot be said of full, deep breathing. It is no hobby or wild notion, but if you would prove its benefits, practice it daily, and you will increase the circulation, purify the blood, and send it rich and hot to warm the feet, make ruby lips, and plant roses on the cheeks. It will aid your digestion, and give you a clean, sweet breath, promote sleep, quiet the nervous system, strengthen the throat and vocal organs, and increase the chest capacity. It will also cure your asthma, catarrh, and bronchitis, and prevent lung trouble."—*Popular Science News.*

